# Dental Literature

**Published:** 1889-10

**Authors:** A. G. Bennett

**Affiliations:** Flushing, N.Y.


### DENTAL LITERATURE.[1]

BY A. G. BENNETT, D.D.S., FLUSHING, N. Y.

YOUR attention is this evening directed to a subject that I presume must be interesting to every lover of his profession. The time and place seem eminently fitting for its presentation and discussion. Modern dental literature is now about fifty years old; the college, the society, and the journal having been founded, organized, and established respectively at about the same time. This city is the dental centre in respect to the number of able and active men, if not as to colleges and journals, and this society is one of the two strongest and most influential organizations in the production of theoretical and practical literature.

I appear before you as one who can testify to the benefits of our literature before attending college or society meetings as well as afterwards, and I take this occasion to express my appreciation of the work you have done in this direction; for many of you I have long known as the authors of valuable contributions, and the participants in interesting discussions, to the benefit of our noble profession.

Even a brief paper on this subject would seem incomplete without some mention of our most advanced thinkers, such as Black and Miller, not to name others; but I have attempted simply to lay the ground-work of the subject, depending on those of you of larger experience to rear the superstructure. With a view to completeness,

---

[1] Read before the First District Dental Society of the State of New York, January 8, 1889.

so far as I go, I have been at times perhaps too elementary, and at others have not escaped the commonplace. Nor would I appear to be magnifying the importance of the periodical; for I am inclined to think that if all dentists were educated as well as they should be, half our present literature as found in the journals would be useless,—would never have existed.

It will be noted that I have illustrated some points and principles, not by absurd or exceptional examples, but by those that may justly be regarded as extreme, and therefore all the more striking.

Dental literature as found in the text-books and journals is a branch of that great department of scientific thought which treats of the diseases of the general system as well as their remedies. It is so intimately connected with preliminary training and subsequent association that its relation to these must first be outlined in a general way. A dental, like a medical, education is the result of three forces or factors: the college, the society, and the journal; and, like a medical education, instead of ending with the commencement, it properly but fairly begins on that eventful day. When the ground-work is laid by the college, the society and the journal and that familiar factor known as experience must be depended on to complete the educational superstructure.

*The College.*—In more senses than one the college is the vestibule to the professional temple, which, in its greater light, gives scope to broader and clearer vision. Far be it from any lover of his profession to belittle the college, even though it does prove to some the "royal road" that leads to learning without labor; or that "other way" through which some climb to a profession, without such credentials as should be required in this enlightened age. It is trite but true that all real progress begins at the beginning, and continues to proceed, if at all, in accordance with the nature of things and in harmony with the constitution of the mind; and I presume all will admit that vigorous growth and professional progress depend almost entirely on the standards of *admission* and *graduation* maintained at the colleges. If these, in their struggle for existence on a business basis, cannot gradually elevate their standards, our investigators and authors labor at a great disadvantage, if not in vain, to enlighten their routinist who looks back on commencement-day as the climax of his mental growth; for it must be conceded that the more thorough the college course, the higher must be the quality of the literature that will be demanded, and, conversely, the lower the standard of admission and graduation, the poorer the quality of our periodicals and the smaller the demand for literature of any kind. The teacher, it has been said, cannot furnish "ready-made" experience, though it is a part of his vocation to reduce for the student the number of lessons that must be learned in that hard school.

Some minds—those that are turned to the light—continue to grow even under the most unfavorable circumstances, others—those who do not have the same affinity for knowledge after a certain period—cease to acquire, and begin to narrow. The one ascends the heights of a broader culture, the other descends into the chill valley of apathetic indifference. The mind that shuts out the light seems to wither rather than to ripen, "but the education of a man of open mind is never ended."

*The Society.*—What is the meaning and value of the society but an organized effort for common improvement? The social feature aside, the object and outcome of such meetings as this is the reading and discussion of papers and the interchange of experience, —in a word, the production of literature. Between the pressure of daily practice and the need of rest, the active members of our societies do much good work. And though the State and national gatherings are, and perhaps must be, in the languid summer vacation, the papers at least give value and interest to some of our leading periodicals. Besides this, the society is in striking contrast to the ancient closeness and narrowness that have not even yet entirely disappeared.

*The Journal.*—The journal is the pioneer of research and investigation, and the transcript of experience. The great and most obvious advantage of all recorded thought is that it removes the barriers of time and space, and brings within our reach all that is most valuable and helpful in the past and the present.

Ever since the days of Bacon, if not long before, the thinkers have partially or entirely turned their attention from the mere appearances of things and have been examining the compositions of organisms and the laws that govern their functions, as well as the causes and remedies for all abnormal conditions. And whilst, on the one hand, such progress has been made that the laity are often led to think that science can settle all things, even to the origin and destiny of man; on the other hand, it must be conceded by the most advanced that very little seems finally or definitely settled. As a proof of this, in dental science itself, the past progress and the present condition of histology need but be mentioned, though without presuming an opinion on its most recent outcome. It has been aptly said that man studies nature, not for the intrinsic value of the knowledge itself, nor for its effect on his mind, but solely for practical purposes. It is not so much that nature wars against man that her forces must be brought under control and guidance, but that the conditions of human life may be made better, the grand end and aim being to make nature the servant of man,—to improve the planet we dwell on, the "house" we dwell in, and thus make life more and more worth the living. And at the very basis of this great work dental science and art find their proper sphere and important position.

The result of nearly all the discoveries in scientific investigation find expression first in the journals.

In general it may be said that the modern periodical is, next to the school, the chief factor and the central feature of progress in all directions. In view of the fact that the humblest pursuits and industries have their periodicals, it seems almost needless that attention should be called to the value and importance of the literature of any profession. It may be claimed that dentistry is largely an art, and that it has been and is still practised as a trade, and that even as a profession it is slightly surgical and largely mechanical; yet the fact remains that the dentist is much more than an educated mechanic; and though he may and should accomplish much in his laboratory, he cannot dispense with his library any more than a physician. The gifted few may depend on their own resources, but the majority must depend largely on the experience of others.

The ideal dentist is a man of thought and action combined. The thoughtless routinist becomes a mere business machine, or, like many an artisan, a part of the machinery he operates. And even the true professional man who has not the leisure for thinking cannot expect much growth. But, as the name implies, the doctor must be a teacher, one who studies that he may give the best instruction to those who seek his services, and that he may be able to reduce to practice his acquirements.

The dental journal should contain not only the best and latest thought of the profession, but at once the most *accessible*, the most *economical*, and the most *universal* means of professional advancement. And though the mission of the journal is to expound principles, discuss methods, and record experience, it must, since it deals in the new and recent, contain considerable of the speculative and the theoretical. But since a theory is merely a *temporary principle*, as many a conclusion is merely a *temporary resting-place*, the so-called practical man must admit the value of whatever is still subject to the tests of criticism and experience. No matter how thorough the colleges may become, the fact remains that dental literature as found in the journals is the chief factor of *post-graduate education.* Hence the essential need of able writers and studious readers. Again, the literature that grows in the journals becomes gradually crystallized into text-books, which in turn become the standards by which the later productions are judged.

It is not the number but the ability of writers that gives value to any and all kinds of literature, especially that of a scientific nature. Some questions seem always open. For example, Dr. Dean, in a recent paper, proceeds to show that all dental legislation springs from mere selfishness, and is solely for the benefit of those in possession of the professional demesne. However debatable this may be, there is no disputing the fact that the chief factor in all progress is *interchange of thought.* Hence the importance of its *quality.*

Dr. Faught, in a paper devoted chiefly to the statistics of literature as represented in one journal (the *Cosmos*), points out the fact that the majority of our writers are or have been connected with the colleges. Why not? The atmosphere of a college is or should be somewhat electrified by scientific thought. There is certainly little in the office, especially in that of a semi-educated man engaged all day in drilling and filling semi-vital organs, to inspire or compel mental activity. Besides, this pursuit is too exhausting. But the college-man is forced to think, and to learn the art of expression, and to have some meaning and method in his discourses. This is true generally, but does not often apply to the professor who from a pressure of practice rushes in at the last moment and rambles all around and over his theme, under the pleasing delusion that the students do not know the difference between a diluted muddle and a solid and systematic effort, with a beginning, a middle, and an end. In the paper just referred to the writer mentions the fact that for almost ten years there were only ten writers from what he calls the ranks, which would indicate dulness or indifference there, or that most of the ability is in the colleges, both of which points are quite debatable. These "ten righteous" may be a discreditably small number, but if they are writers of the first order they should, even now, be able to save the profession from the danger of being inundated by the annual outpouring of new-recruits.

1. *The Writer.*—As already implied, the colleges must train our thinkers as well as do no small part of our thinking, there being no good reason why their direct influence should cease with the commencement. But the nature of dental art is such that all our thinkers need not be writers. The man with a genius for invention and a talent for the artistic will find full scope for his best endeavors. Some can best express themselves by the product of their hands, an art that cannot be well taught by words, and one that is, after all, the most effective means of expressing an idea. How important it is to possess that artistic sense, and perceive truly the meaning of *form*, which is said to hold a middle place between matter and mind, moulding the one and expressing the other! And instances are not wanting when the original work of one can be best expressed in the words of another. Emerson says, "Each man of thought is surrounded by wiser men than he, if they cannot write as well. Why cannot he and they combine?" Again, the compiler is one of the most useful writers in every department of knowledge. Next to the investigators who work and write largely for the most advanced, we as a profession need men who have the faculty and industry to write clear, concise, and systematic summaries of our annual progress in every direction. And if an editor can give the essence of some controverted point or subject every few months, so much the better for all concerned. Why the compiler is needed in dentistry ought to be obvious enough. The practice of dentistry is so exacting and exhausting that the busy or weary man must often take his reading in a condensed form; and the lazy man takes his in that shape or not at all. And, in general, the pressure and haste of modern life demand that the attention be not unduly taxed by mere phrases or a vain parade of words, that serve only to conceal poverty of thought. Again, in the course of time ideas and theories and devices that filled much space in journals and books are compressed into a few paragraphs or sentences, and even into clauses and words. Were it otherwise, practical professional literature would be buried beneath a mass of mere verbiage, and become inaccessible to the many who need it most. Besides, after becoming more or less familiar with the ideas that belong to any subject, the discriminating reader seeks their best form, their most classical expression. But an article or a book on a new subject must be something more than a list of practical methods. This applies particularly to any subject that has not passed the stage of controversy. The principles or the theory at least should be laid down in clear and systematic outline. Something more than mere dogmatism must be demanded, or the writer must not be surprised at the stereotyped reply, "I don't believe in it; it is all wrong." Since methods occupy so much space in dental literature, good descriptive writers are always in demand. One who can by a suggestive word or a felicitous phrase make things "live in description" has a kind of genius that always will be appreciated. And he who can embody a thought in a striking figure of speech has the most effective means of arresting attention and exciting interest. But it is not claimed that a complicated subject can be made clear and simple, even by the most expert writer, without the reader's giving the aid of his thinking.

It may be asked here, What are the claims of style in scientific writing, which primarily aims simply at precision and clearness? It is trite to say that style is dress, but it is more; it is manner, which counts for almost everything. That it has some claims no one can deny. The cultivated must not be repelled, and the semi-educated must be attracted; for even those who have the most intense curiosity about things, scientific and artistic, are not proof against the effect of a loose, rambling style, that has neither meaning nor method. Scientific truth finds expression more frequently in an Emersonian simplicity than in an Addisonian elegance of diction. A harsh and halting style repels. Take this instance: A well-educated student was advised to read a certain section in a recent work on dentistry, and though the subject is one of the most absorbingly interesting and practical of all, the reader was repelled by the writer's labored efforts to present simple things in a learned style. Many readers have a similar experience, without perhaps being able to name the cause. A bad style is a worse non-conductor between the writer and the reader than a manuscript is between a speaker and his audience. Some things do not always get a hearing as soon as might be expected. May not this be owing, in part at least, to the manner of their presentation? Again, points that are suggestive, that awaken thought, are often much more valuable than many of the so-called practical points that give no hint of the *why* and *when.*

A slight acquaintance at least with the masters of thought and expression will not detract from one's ability as a filler of teeth. The reader may not be impressed with the simple majesty of Milton, and he may be repelled by the pompous dignity of Johnson; he must, however, be blind indeed if he misses the terse force compressed into the metrical essays of Pope. If he seeks the master in the art of expression, he can find none so clear and forcible as to details, none so comprehensive as to generalities, as Herbert Spencer. This writer takes the world of mind and matter for his province, and no subject is so profound or so complex as to elude his broad philosophic insight or interfere with a vigorous and systematic exposition. He has that rare combination of a good command of language and a better command of ideas.

*The Reader.*—Burke says, "He that borrows the aid of an equal understanding doubles his own." However that may be, it seems to me that a man may better be known by the books he reads than by the company he keeps, for the obvious reason that the choice of the former is always free.

In order that we *know more* and *do better*, we must have good writers; but what we need most of all is a larger number of *studious readers.* It is of course assumed that the colleges develop that degree of intelligence which is needful to understand and appreciate the best literature. Reading alone will not suffice; but given a better training, it is claimed that more persistent efforts at self-improvement will greatly reduce the difference that exists to-day between the routinist who reads little and thinks less, and the progressive practitioner who, though he may not be original in any direction, is well-read in every branch of his profession. But what of the man who has never seen a college? Enforce the laws, and by and by that question will answer itself.

We may need better journals, but create a demand for a higher literature and the supply will take care of itself. According to some statistics, already referred to, furnished by Dr. Faught, it seems that in almost ten years—1872 to 1881—ten writers did nearly all the literary work in the *Cosmos* outside of the colleges, which mustered about fifteen in the same period. It seems to me that there are more significant statistics, though perhaps more difficult to obtain. For instance, this is a pertinent point: How many dentists made our literature a study? Of these, how many are graduates? How many men at the end of each year will stand a good examination on all that has any value in the new books and two or three of the best journals? These questions may be more suggestive than answerable, but I think them quite pertinent.

When we have enough studious readers, our journals need not be published at the depots. Given the readers, a journal published by dentists and for dentists never need "perish from the earth." Yet a journal may die because it is too good to live, or fails to strike the average intelligence. But we do not begin at the beginning. Dental history is not taught in the colleges, and the periodicals are only incidentally mentioned. The college instructor who boasted of the schools he attended, and sneered at those who write for the journals, but who neither wrote, nor perhaps read, may fitly represent the semi-educated man who thinks the text-books of some years ago final and all-sufficient.

Bacon's familiar saying, to the effect that reading, talking, and writing will make respectively a full, ready, and exact man, is in point. This is made complete by Hobbs's saying that if he read as much, and thought as little, as some of his friends, he would be as ignorant as they. Reading alone is certain to produce *congestion of ideas*, but thinking, talking, and writing result in perfect assimilation.

What, then, constitutes the ideal reader? He whose training has given him the key to art, science, literature, and philosophy.

Dental literature, like all that is scientific, requires that the reader give the assistance of his thinking to what he reads; for, says Dr. Mitchell, the best readers are, in a measure, co-operative authors, and may be left to interpolate the unsaid. It has been well stated that it is an interesting problem for a man to solve, how far he shall profit by the thoughts of other men and not be enslaved by them. Can it be that the fear of being enslaved keeps down the subscription-lists of our journals? I hope not. Only the few great minds of our race,—those that have been illumined by a spark of that immortal fire called genius,—only these can create a world of thought that is all their own. Yet even these must have their materials, which must be gathered by more tangible means than inspiration. An English essayist says that each one, as he steps on the stage of action, has placed at his disposal the accumulated mental capital of the ages. Yet in one sense this capital is stored in mines and cannot come into his possession except at the cost of much labor. The fields of thought are sown, but each must reap and garner the harvest for himself.

The same writer says that what a man has learned is of importance, but what he is, *what he can do*, what he will become, are more significant things. Reading, as well as thinking, to have any value, must be undertaken with a definite object and with studious care. That "definite object" means self-improvement, and it should also mean *aid to others.* One who reads carefully and takes notes, and then amplifies them so that they have meaning and method, has done much to fix what may have been a cloudy subject clearly in his mind. And if he tries to gather the real gist from a prolix or diffuse writer, or from one who still retains his sophomoric floridity of diction, he will be surprised at the small amount of wheat buried under the mountains of chaff. A man need not write for print alone, but to review his knowledge and to fix it firmly in his mind; for a man's learning that is not in constant use never seems so little as when he draws his pen for a grand review on paper. He is surprised at the "absentees" and the straggling appearance his ideas make when they answer to the mental roll-call. A practical point here is that papers to be discussed at society meetings should be announced always a month in advance, in order that each can so prepare as to elevate this kind of literature.

How much failure is directly due to the lack of what one could readily learn from books and journals is one of those questions that is more suggestive than answerable. Let me give you two significant instances from general and special surgery, which is the most tangible branch of practice. The most marked advance in modern surgery has been made by antiseptics introduced twenty-five years ago by Professor Lister. Just previously, this distinguished surgeon had lost, during three years, about forty-five per cent. of his cases, chiefly from septic diseases. During the next three years, while his methods were still crude, the mortality fell to fifteen per cent. Having perfected his system, he treated during sixteen years five hundred and fifty-three grave surgical cases, with a mortality from septic disease of only one-third of one per cent. This was all done by the same man, for same kind of injuries, in same class of patients. Professor Lister and his colleague, Mr. Spence, who declined to use antiseptics, worked in this same London hospital, and the results show that Spence lost three times as many patients as Lister, while the deaths from infective diseases were eight times greater among the patients of the former than among those of the latter. More striking records than these could be given, but this will suffice. Such and so great is the difference between the progressive and conservative practitioner, between the man whose mind is always open and turned to the light, and the man who follows the old ways, as if all were known, or nothing more was needed.

Take an instance from practical dentistry. In the *Cosmos* for August, 1884, Dr. Atkinson gives the case of an abscess which, before coming into his hands, had been under treatment for five years, and almost all this time by the same dentist. It was an ordinary abscess on an upper incisor, and hence within easy reach; the methods used—that of opening through the tooth and process with a drill and applying peroxide of hydrogen—were known, at least, to all progressive men, and could have been known and applied by the dentist who first had charge of the case. The doctor had not only the chronic abscess to treat, but had to counteract much overtreatment. Here is food for thought. What are the lessons? As Dr. Atkinson suggests, they are briefly these: first, attend society meetings and consult with others of more experience; second, know the *how* and *why* of *what* you are doing; third, trust to nature more and over-treat less; fourth, practise less in a commercial and more in a professional sense.

When any operation, especially a difficult, doubtful or unusual one, is performed, it is well to ask this question, Is this the best as to principle, method, or remedy that modern dentistry can do for this case? This may not at once make one more skilful, but it will awaken thought, and invite comparison that may not always be very flattering.

*The Critic.*—The proper function of the critic is to weigh evidence and to establish truth. Of critics in general it may be said that not being of an investigating or inventive turn of mind, they study the works of others, in order to determine the intrinsic value and actual utility of all that constitutes the fund of knowledge in any branch of learning. It is true that, strictly speaking, we have no class of men who specially attend to this kind of work; yet every true student of science is in some sense a critic.

It would seem that if critics are needed in the sphere of taste, which within certain limits is always changing, how much more are they required in matters of fact and questions of science, which are fixed and final! Any one can suggest a doubt or raise an objection; he alone is the true critic who, while perceiving imperfections, can remove them or indicate a remedy. The mere fault-finder becomes captious and querulous, and the partisan becomes personal. Any profession that depends on science as a basis must hold its investigators to strict account, since, from the nature of things, but few can verify or disprove their supposed facts or alleged principles. Two little questions cover the ground: Is it true? as to science. Is it practical? as to art. What if some subjects of no mean calibre, when exposed to scientific tests, present almost microscopic dimensions, or that others, that met with a cold reception, grow rather than shrink under discussion. It is safe to say that no idea or device of real value was ever yet forced from the field by intolerant criticism. Every new thing must be closely scrutinized and may be even sharply criticised; yet, it may be justly asked by what standard is a new system or appliance to be judged? Certainly not entirely by experience; nor is it to be accepted or rejected on general principles or abstract reasoning. It is related of the distinguished inventor, Sir Joseph Whitworth, that when anything new occurred to or was shown him, he did not proceed to argue one way or the other, but always said, "Let us try it." Many a reputation for sagacity is often risked, and frequently lost, because new ideas and devices of any value persist in having their claims allowed, and insist on having a future. It is well to note in passing that we generally judge new things by old and fixed standards which often have taken a long time to adjust.

There are two elements, the progressive and the conservative, that are always contending for the mastery, both of which are essential to substantial progress. The observing and inventive mind sees too much imperfection to be satisfied with present attainments; the conservative who has ceased to acquire has not the attitude of mind to inspect the new or re-examine familiar things that he has long regarded as fixed and permanent. The progressive man sighs for new kingdoms to conquer; the conservative seems to be satisfied, apparently because he has possession of the field. The progressive man, dazzled by the promise of the present, fails to profit by the lessons of experience; the conservative naturally clings to the past, and, along with much of value, retains many mildewed ideas that have perhaps served as stepping-stones here and there in the paths of progress. In the youthful mind, "too inexperienced to reason, too buoyant to doubt," the new idea finds a fertile soil that produces rapid growth; in the old the soil is more or less barren, and ideas take root slowly. But matters of opinion are largely points of view, and changing this seems to be a difficult matter, even with the most enlightened. Some seem by their attitude to say, "We have enough, there can be nothing new." We have, however, no fear of a limit being set to scientific progress.

I will close by quoting what I consider one of the soundest axioms in dental literature and practice, and to emphasize the necessity of looking at both sides of a subject: "The operator must be guided by a judgment based, not on the questionable work of one, but on the carefully collated knowledge of many."

